# P-124. Ongoing Infections with Extensively Drug-Resistant *Campylobacter jejuni* Linked to Pet Store Puppies

**DOI:** 10.1093/ofid/ofae631.329

**Published:** 2025-01-29

**Authors:** Louise Francois Watkins, G Sean Stapleton, Lavin Joseph, Christy Bennett, Grace Vahey, Katharine Benedict, Laura A Cooley

**Affiliations:** Division of Foodborne, Waterborne, and Environmental Diseases, Centers for Disease Control and Prevention, Atlanta, Georgia; Centers for Disease Control and Prevention, Atlanta, Georgia; Centers for Disease Control and Prevention, Atlanta, Georgia; Centers for Disease Control and Prevention, Atlanta, Georgia; Centers for Disease Control and Prevention, Atlanta, Georgia; Centers for Disease Control and Prevention, Atlanta, Georgia; Centers for Disease Control and Prevention, Atlanta, Georgia

## Abstract

**Background:**

*Campylobacter* is the leading cause of enteric bacterial infections in the United States, causing an estimated 1.5 million illnesses annually. Antimicrobial therapy can reduce the duration of illness and limit complications; macrolides and fluoroquinolones are typically recommended as first- and second-line treatment, respectively. The Centers for Disease Control and Prevention (CDC) investigates emerging resistance in *Campylobacter* strains to identify potential sources and collaborate on approaches to reduce its spread. Here, we describe extensively drug-resistant (XDR) strains linked to pet store puppies.

**Methods:**

State public health departments perform whole genome sequencing on *Campylobacter* isolates as resources allow and submit sequence data to CDC. CDC assesses isolate relatedness using core genome multilocus sequence typing (cgMLST) and screens isolate sequences for resistance determinants to predict antimicrobial resistance (older isolates sequenced retroactively are also screened). We considered isolates to be XDR if they showed predicted resistance to macrolides, fluoroquinolones, and ≥ 3 additional antibiotic classes. Persons with XDR isolates were interviewed about exposures when possible.

**Results:**

We identified three strains of XDR *C. jejuni*; the oldest isolates were from 2007. Although these strains are not highly related phylogenetically ( > 200 allele differences between strains by cgMLST), cases share epidemiological and clinical characteristics. As of June 30, 2023, CDC received reports of 274 cases associated with these strains. Affected individuals had a median age of 36 years (interquartile range, 20–52); 66% were female, and 23% were hospitalized. Of those affected, 97% reported contact with a dog before becoming ill, among whom 78% reported contact with a puppy from a pet store.

**Conclusion:**

Clinicians should be aware that strains of XDR *Campylobacter* are circulating in the United States and should consider XDR infection when patients fail to respond to routine treatment, particularly if they have recently had contact with pet store puppies. Carbapenem antibiotics may be used for severe infections that are resistant to recommended treatment agents.

**Disclosures:**

**All Authors**: No reported disclosures

